# Willingness to provide behavioral health recommendations: a cross-sectional study of entering medical students

**DOI:** 10.1186/1472-6920-12-28

**Published:** 2012-05-08

**Authors:** Stephen A McCurdy

**Affiliations:** 1 Regional Medicine-Public Health Education Center, Department of Public Health Sciences, University of California, Davis School of Medicine, One Shields Ave., MS-1C, Room 181, Davis, CA, 95616-8638, USA

**Keywords:** Behavioral health recommendations, Medical student counseling, Adolescent sexuality, Sexual abstinence

## Abstract

**Background:**

Behavioral factors contribute importantly to morbidity and mortality, and physicians are trusted sources for information on reducing associated risks. Unfortunately, many clinical encounters do not include prevention counseling, and medical school curriculum plays an important role in training and promoting such counseling among medical students.

**Methods:**

We surveyed all 93 freshman medical students at entry to the University of California, Davis School of Medicine in 2009 to evaluate baseline knowledge of population health principles and examine their approach to clinical situations involving four common behavioral risk factors illustrated in brief clinical vignettes: smoking, alcohol use in a patient with indications of alcoholism, diet and exercise in an overweight sedentary patient, and a 16-year-old contemplating initiation of sexual intercourse. Based on vignette responses, we assessed willingness to (1) *provide information* on risks, (2) *recommend elimination of the behavior* as the most efficacious means for reducing risk, (3) *include strategies apart from elimination* of the behavior for lowering risk (i.e., harm reduction), and (4) *assure of their intention to continue care* whether or not recommendations are accepted.

**Results:**

Students answered correctly 71.4 % (median; interquartile range 66.7 % - 85.7 %) of clinical prevention and population health knowledge questions; men scored higher than women (median 83.3 % vs. 66.7 %, p<0.02). Students showed high willingness to provide information and strategies for harm reduction apart from risk elimination, while respecting patient autonomy. Willingness to recommend elimination of high-risk behaviors “always or nearly always” was high for smoking (78.5 %), alcohol consumption in a patient with indications of alcoholism (64.5 %), and diet and exercise in a sedentary and overweight individual (87.1 %), and low for the 16-year-old considering initiating sexual intercourse (28.0 %; Friedman test, p<0.001). Willingness was not associated with the respondent’s background knowledge of population health principles or gender.

**Conclusion:**

Students showed high willingness to educate and respect patient autonomy. There was high willingness to recommend elimination of risk behaviors for smoking, alcohol, and poor diet/exercise, but not for sexual intercourse in an adolescent considering sexual debut. Further research should address promoting appropriate science-based preventive health messages, and curriculum should include explicit discussion of content of recommendations.

## Background

Behavioral and lifestyle factors exert important influence on health, and in the US approximately half of deaths are linked to preventable behavioral and social risk factors, with smoking and poor diet and exercise as leading causes [1]. Physicians have historically been trusted sources of information and recommendations regarding associated health risks and their mitigation. Ideally, preventive health recommendations should be clear, science-based, and artfully proffered so as to respect individual autonomy. The Guide to Community Preventive Services [2] and the U.S. Preventive Services Task Force’s Guide to Clinical Preventive Services [3] are useful resources for evidence-based prevention measures on various health topics. Both include an assessment of the underlying science for each prevention measure, ranging from “insufficient evidence” to “recommended.” Professional and community organizations, such as the American Cancer Society, American Academy of Pediatrics, Alcoholics Anonymous, and others also provide educational materials and preventive health recommendations, although these are not always evidence-based. In the clinical setting, physicians have the opportunity to provide prevention messages directly to patients, yet many clinical encounters do not include prevention counseling [4,5].

Medical school curriculum plays an important role in training students to discuss behavioral health and lifestyle risks with their patients. Factors associated with prevention counseling and its perception as important among U.S. medical students include interest in prevention and a primary care career, female sex, non-White ethnicity, healthy personal practices, and attending a medical school that encourages healthy lifestyle for its students [6]. However, most studies focus on perceived relevance and frequency of prevention counseling and do not explicitly address its substance beyond identifying the topic. Thus, there is little published information on whether counseling is limited to a neutral discussion of the risks and benefits of the courses open to the patient (i.e., continuing with, mitigating through reduction or other means, or eliminating an unhealthy practice) or also includes a recommendation for one of the possible courses of action.

This study aimed to assess prevention knowledge among entering first-year medical students and characterize their approach to providing preventive behavioral health counseling, as indicated by their responses to brief clinical vignettes illustrating common behavioral health risk factors: smoking; alcohol consumption in a patient with indications of alcoholism; diet and exercise in an overweight, sedentary individual; and adolescent sexual activity. The study examined willingness to engage in four aspects of health communication: (1) providing information on risks associated with the health behavior, (2) recommending elimination of the risk factor as the most efficacious means for reducing risk, (3) including alternative strategies for reducing risk apart from eliminating the risk factor (i.e., harm reduction), and (4) assuring patients they would continue as their physician whether or not recommendations were accepted (i.e., respect for patient autonomy).

## Methods

### Student population

The University of California, Davis School of Medicine, one of five medical schools in the ten-campus University of California system, is nationally ranked among the top 20 schools for primary care training and the top 50 schools for research [7]. Students in the 2009 entering class had a mean undergraduate grade-point average of 3.57 and mean Medical College Admission Test total scores of 31.7. Comparable figures for all U.S. medical school matriculants in 2009 are 3.66 and 30.8, respectively [8].

### Survey

First-year medical students entering the School of Medicine in Summer 2009 completed an anonymous self-administered paper survey addressing knowledge and attitudes relevant to public health. We administered the survey during the students’ initial welcome and orientation session, leading to a 100 % response rate. There were 19 knowledge questions representing the knowledge domains of the Clinical Prevention and Population Health Curriculum Framework developed by the Healthy People Curriculum Task Force convened by the Association of Teachers of Preventive Medicine and the Association of Academic Health Centers [9]. To assess willingness to provide preventive health information and recommendations, the survey also included four clinical vignettes (Table 1), each illustrating a common behavioral health risk factor: a 45-year-old smoker; ongoing alcohol consumption in a 38-year-old with history suggestive of alcoholism; a sedentary and overweight 23-year-old; and a 16-year-old contemplating becoming sexually active. Sex and ethnicity for the vignette patients were not provided.

**Table 1:** Clinical vignettes

**“How would you advise . . .**	**Elements of counseling**
. . . a 45-y.o. smoker?”	1) Discuss risks of smoking
	2) Make a clear recommendation for quitting smoking
	3) Discuss ways to reduce risk if smoking continues (e.g., reduce consumption)
	4) Assure patient you will care for him or her regardless of his or her decision regarding quitting smoking
. . . a 38 y.o. with signs of alcoholism, including several “driving while intoxicated” arrests?”	1) Discuss risks of alcohol
	2) Make a clear recommendation for abstinence from alcohol
	3) Discuss ways to reduce risk if drinking continues (e.g., limit consumption, avoid driving when drinking)
	4) Assure patient you will care for him or her regardless of his or her decision regarding cessation of alcohol use
. . . a 23 y.o. who is sedentary and 20 pounds overweight?”	1) Discuss risks of sedentary lifestyle and obesity
	2) Make a clear recommendation for improved diet and exercise
	3) Discuss ways to reduce risk if the patient does not wish to change diet or exercise (e.g., suggest small changes)
	4) Assure patient you will care for him or her regardless of his or her weight and decision regarding diet and exercise
. . . a 16 y.o. contemplating becoming sexually active, including intercourse?”	1) Discuss risks of sexual intercourse
	2) Make a clear recommendation for abstinence from sexual intercourse
	3) Discuss ways to reduce risk if the patient begins engaging in sexual intercourse (e.g., condom use, contraception)
	4) Assure patient you will care for him or her regardless of his or her decision regarding sexual activity.

^a^ Students answered the four questions for each vignette using a five-level Likert scale addressing likelihood of providing the designated information: (0) never, (1) rarely, (2) about half of cases, (3) usually, or (4) always or nearly always.

The survey used a five-level Likert scale to indicate willingness in each vignette to provide information or recommendations in four areas of communication: (1) *provide information* on associated risks, (2) *recommend elimination* of the behavioral risk factor as the most efficacious means for reducing risk, (3) include *alternative strategies* apart from risk factor elimination for lowering risk (i.e., harm reduction), and (4) *assure patients* of their intention to continue care whether or not recommendations are accepted. The Likert categories and their associated numeric values were “never” (0), “rarely” (1), “about half of cases” (2), “usually” (3), and “always or nearly always” (4). For each of the four areas of communication, we calculated an average willingness score based on the numeric values zero through four from the Likert scale. To maintain brevity and reduce the time demand for completing the survey, each individual questionnaire contained approximately one-third of the 19 knowledge questions and all of the four clinical vignettes.

### Statistical analysis

Analyses were performed using the Stata IC statistical package, version 11 (College Station, TX). Population health knowledge is presented as percent of questions answered correctly. Group comparisons for knowledge scores were evaluated with the Wilcoxon rank sum test [10]. The Spearman correlation described the association between percent of knowledge questions correctly answered and the willingness scores described above [10]. Friedman’s rank test assessed whether willingness scores were similar across the four cases within each of the four areas of communication [11]. Post-hoc pair-wise comparisons, adjusted for multiple comparisons, identified which case’s scores were significantly different if an overall difference across the cases was found. The University of California, Davis Institutional Review Board approved the study.

## Results

### Demographics

All 93 members of the entering medical school class completed the survey; the median age of respondents was 25.0 y (interquartile range 23.7 – 26.7 y). Compared to the national population of entering U.S. medical students [8], School of Medicine students were more likely to be women (59.1 % vs. 48.3 %) and Asian or Pacific Islander (36.6 % vs. 22.7 %) or Hispanic (12.9 % vs. 7.9 %) and less likely to be White (46.2 % vs. 65.1 %) or African-American (4.3 % vs. 7.5 %).

### Population health knowledge

Students answered correctly 71.4 % (median; interquartile range 66.7 % - 85.7 %) of the clinical prevention and population health knowledge questions. The lowest scores (<50 % correct) were for interpreting p values and for knowledge of viral influenza chemoprophylaxis. The highest scores (﹥95 % correct) were for questions related to the public health system and health communication. Men achieved higher average knowledge scores than women (median 83.3 % vs. 66.7 %, p﹤0.02, Wilcoxon rank sum test).

### Willingness to provide behavioral health information, recommendations, and assure continued care

Students showed high and statistically similar levels of willingness to provide information on associated health risks for the behavioral risk factors illustrated in the four vignettes; similar results were seen for willingness to provide alternative strategies for lowering risk apart from risk factor elimination (i.e., harm reduction) and willingness to assure patients of their intention to continue as their physician whether or not recommendations are accepted (Table 2). Of the four clinical vignettes, students showed the greatest willingness to discuss health risks, provide harm reduction information, and assure that they would continue as the patient’s physician in the case of the 16-year-old contemplating initiating sexual intercourse.

**Table 2:** Willingness of entering first-year medical students to provide behavioral health information and recommendations

**Areas of communication**	**Intention to provide information “always or nearly always” for the indicated risk factor [(% of respondents)Average willingness score**^**a**^**]**			
	**Smoking in 45-year-old**	**Alcohol use in 38-year-old with indications of alcoholism**	**Diet and exercise in overweight, sedentary 23-year-old**	**Sexual activity including intercourse in 16-year-old**
Discuss associated health risks	(90.3 %)	(86.0 %)	(80.7 %)	(95.7 %)
	3.87	3.73	3.80	3.95
Recommend elimination of high-risk behavior	(78.5 %)	(64.5 %)	(87.1 %)	(28.0 %)
	3.73	3.40	3.83	2.11^*^
Provide harm reduction information	(79.6 %)	(85.0 %)	(74.2 %)	(94.6 %)
	3.75	3.76	3.66	3.94
Assure patient of continued care whether or not recommendations are accepted	(73.1 %)	(71.0 %)	(78.5 %)	(90.2 %)
	3.61	3.54	3.66	3.84

^a^ Average willingness score based on a five-level Likert scale addressing the respondent’s self-reported likelihood of providing the information subset: (0) never, (1) rarely, (2) about half of cases, (3) usually, or (4) always or nearly always.

* p﹤0.001, Friedman’s rank test for comparison of willingness scores across the four clinical scenarios. Pair-wise comparisons, adjusted for multiple comparisons, revealed that this result was statistically different from the willingness scores in this area of communication for the other cases.

Willingness to recommend risk factor elimination was highest for poor diet and lack of exercise in the overweight, sedentary individual, followed by smoking and alcohol in a patient with signs of alcoholism. Only 28 % of students were willing “always or nearly always” to recommend sexual abstinence to the 16-year-old patient, and 15.1 % indicated they would “never” recommend sexual abstinence in this situation. In contrast, no student indicated he or she would “never” recommend risk factor elimination in the case of smoking and the sedentary, overweight individual, and 2.1 % of students indicated they would “never” recommend risk factor elimination in the patient with signs of alcoholism. Willingness to recommend risk factor elimination was similar for smoking, alcohol, and diet and exercise, and statistically significantly lower for adolescent sexual activity including intercourse (Friedman’s rank test; p﹤0.001).

Clinical prevention and population health knowledge score and respondent sex were not correlated with willingness to provide information on risks associated with the health behavior, to recommend risk factor elimination as the most efficacious means for reducing risk, to discuss alternative strategies for reducing risk apart from eliminating the risk factor, or to assure patients they would continue as their physician whether or not recommendations were accepted.

## Discussion

Harms associated with the vignette topics illustrated here are well known, and our findings suggest that this common knowledge is reflected in the willingness of entering medical students to educate regarding these behavioral risk factors. Students also showed high levels of respect for patient autonomy, as indicated by willingness to assure patients of continued care whether or not the patient accepted the proffered health recommendations. With respect to recommending risk factor elimination, students were most willing to recommend elimination of diet and exercise risk factors in an overweight and sedentary individual, followed by smoking and continued alcohol use in a patient with indications of alcoholism. The most marked (and statistically significant) finding relates to the adolescent contemplating initiation of sexual intercourse: only 28 % of students were willing “always or nearly always” to recommend avoidance of sexual intercourse, and 15.1 % indicated they would “never” recommend abstinence in this context. Neither respondent gender nor population health knowledge score affected willingness to educate, offer preventive advice, or respect patient autonomy.

Health professionals have historically relied on scientific information to craft educational messages and make recommendations. There are many examples in addition to those illustrated in this study: rather than simply providing facts about seat belts, speeding, fire safety, and other health topics involving behavioral health risk factors, health professionals have taken the additional step of making clear recommendations based on the underlying science. Accordingly, health professionals should be comfortable in providing science-based information and recommendations regarding adolescent sexual activity, as with other behavioral health risk factors.

The most notable adverse consequences of adolescent sexual activity include unintended pregnancy (approximately 650,000 for U.S. women﹤20 years of age in 2006 [12]) and sexually transmitted diseases (e.g., 420,101 new cases of *Chlamydia* infection among U.S. 15–19 year-olds in 2008 [13]). Although not necessarily causally related, adolescent sexual activity is also associated with emotional ill health [14]; use of tobacco, alcohol, and illicit drugs [15,16]; and low academic achievement [17] with negative socioeconomic consequences in later life. Because of their developmental, social, and financial state of maturity, adolescents are generally less able than independent adults to deal with the adverse consequences of sexual intercourse should they occur. Contraception, condoms, and other means can mitigate the risks for unintended pregnancy and infection, but there is no disagreement that abstinence is the most efficacious preventive measure [18-22]. Thus, reluctance to proceed beyond providing information to recommending against sexual intercourse in this age group appears inconsistent with practice standards for other behavioral health risk factors and with data on associated harms.

A large national survey of U.S. medical students documents unwillingness to limit sexual health education to an abstinence-only message and a preference for comprehensive approaches including alternative strategies for reducing risk, such as cautious selection of partners, contraception, and condom use [23]. Yet counseling about alternative strategies for risk reduction need not exclude a recommendation of abstinence from sexual intercourse as the most efficacious means of prevention. Medical students and physicians can educate adolescent patients who are considering becoming or already are sexually active about strengths and limitations of available means of prevention and also recommend abstinence, emphasizing that the recommendation is grounded not in moral condemnation, but in concern for protecting their health, and that the physician will continue to care for the patient whatever their decision.

Some may argue that a recommendation for sexual abstinence is unlikely to be heeded and may alienate adolescents. Yet research suggests that adolescents appreciate honest and non-judgmental discussions with health care professionals [24]. Low acceptance rates for recommendations to stop smoking, refrain from inordinate alcohol consumption, and obtain proper diet and exercise have not deterred health professionals from making artful and respectful science-based recommendations without alienating patients or communities.

Medical school curricula for behavioral health vary widely in form and content, and the topic poses many pedagogical challenges [25]. In the relatively noncontroversial case of smoking, guidelines are available that include a clear recommendation for smoking cessation or avoidance [26]. Yet for fraught subjects such as sexuality, there is disagreement in society—reflected here among our entering medical students—about content of such recommendations.

At the University of California, Davis, behavioral health recommendations arise naturally in the clinical setting and are also addressed in the longitudinal Doctoring course spanning the four-year curriculum [27]. In Doctoring small-group sessions, students discuss cases and interview standardized patients, providing the opportunity to address behavioral health recommendations. Whereas students discuss recommendations for the individual cases, there is at present no overarching discussion addressing underlying principles of determining the content of recommendations. Such a discussion may not lead to full consensus on content, especially for controversial subjects such as sexuality, yet should spur thinking and a mindful, rather than automatic, approach to the patient.

Important strengths for this study include its setting in a highly ranked U.S. medical school, high response rate (100 %), and focus on the substance of counseling offered by medical students as reflected in clinical vignettes for common clinical problems. The study has three important limitations. First, it is set in only one of the more than 150 accredited schools of medicine or osteopathic medicine in the U.S. University of California, Davis School of Medicine students were more likely to be women and Asian or Hispanic than the national population of entering medical students in 2009, yet they had similar mean grade-point average and Medical College Admission Test scores.

It is possible that the different demographic characteristics of the students compared to the national population of U.S. medical students affected our results. For example, a large national study of U.S. medical students showed that women and non-Whites—groups over-represented in our students compared to nationally—were more likely to report counseling among general medicine patients [6]. However, the magnitude of the differences in counseling frequency scores between groups was small—approximately 5 % between men and women and less than 10 % between the various ethnic groups comprising the respondents. Although we did not collect information from the respondents on ethnicity on our survey, respondent gender had no bearing on likelihood of recommending elimination of risk factors. Thus, it is likely that School of Medicine students and these results reflect a national rather than a regional perspective with respect to willingness to make behavioral health recommendations.

Second, the study focused on entering medical students, and responses represent intention based on their education, values, and experience prior to beginning the medical curriculum. Although the students’ approach to counseling patients regarding behavioral risk factors may change as they progress through medical school and into practice, it is likely that the attitudes they bring at entrance will be influential, and medical school educators should be aware of this as they design relevant curriculum.

Third, the clinical vignette format unavoidably imposes limitations that may affect response. For example, willingness to provide information and recommendations may vary according to the patient’s sex, perceived maturity, presence of other medical conditions, degree to which the patient is known to the caregiver, and circumstances of the clinic visit, none of which were indicated in these vignettes. While these factors may have affected overall willingness to provide information and recommendations, it is unlikely that they explain the marked reluctance to recommend against sexual intercourse in adolescents compared to the behavioral risk factors illustrated in the other three vignettes. This reticence may result from cultural characteristics attendant to the students’ early stage of professional development, a belief that sexual activity among adolescents carries only rare and inconsequential risks, conviction that making recommendations in this area is inappropriate or futile, or to personal discomfort with the topic.

## Conclusion

Physicians are trusted sources of health-related information and advice. It remains the patient’s decision as to whether to heed that advice, but this does not mean that the physician becomes simply a neutral source of information, unwilling to recommend, as part of the shared decision-making conversation [28], that which science suggests is in their best health interest. Students showed high willingness to educate and respect patient autonomy. We observed high willingness to recommend elimination of risk behaviors for smoking, alcohol, and poor diet/exercise, but not for sexual intercourse in an adolescent. Further work should include research into understanding correlates of willingness to engage in preventive health counseling by health professionals and students, including effect on message content, and on improving skills and attitudes for promoting science-based health recommendations in a respectful and effective manner. Medical curriculum should include explicit discussion of content of recommendations, especially for fraught subjects such as sexuality where consensus may not occur, to promote thinking and a mindful approach to health promotion.

## Competing interests

The author has no competing interests.

## Pre-publication history

The pre-publication history for this paper can be accessed here:

http://www.biomedcentral.com/1472-6920/12/28/prepub

## References

[B1] McGinnis JM Foege WH Actual causes of death in the United States JAMA 1993 270 18 2207 2212 10.1001/jama.1993.03510180077038 8411605

[B2] Stephanie Zaza , Briss PA, Harris KW Guide to Community Preventive Health Services 2005 Oxford University Press, Oxford, UK

[B3] Agency for Health Care Research and Quality The Guide to Clinical Preventive Services, 2009: Recommendations of the U.S. Preventive Services Task Force 2009 Department of Health and Human Services, Agency for Healthcare Research and Quality, Washington, DC

[B4] Frank E Harvey LK Prevention advice rates of women and men physicians Arch Fam Med 1996 5 4 215 219 10.1001/archfami.5.4.215 8769910

[B5] Reed MB Burns DM A population-based examination of racial and ethnic differences in receiving physicians' advice to quit smoking Nicotine Tob Res 2008 10 9 1487 1494 10.1080/14622200802323217 19023840

[B6] Frank E Carrera JS Elon L Hertzberg VS Predictors of US medical students' prevention counseling practices Prev Med 2007 44 1 76 81 10.1016/j.ypmed.2006.07.018 16978687

[B7] Best Medical Schools http://grad-schools.usnews.rankingsandreviews.com/best-graduate-schools/top-medical-schools

[B8] Vassev P, Geraci W AAMC Data Book: Medical Schools and Teaching Hospitals by the Numbers 2010 Association of American Medical Colleges, Chicago, IL

[B9] Allan J Barwick TA Cashman S Cawley JF Day C Douglass CW Evans CH Garr DR Maeshiro R McCarthy RL Clinical prevention and population health: curriculum framework for health professions Am J Prev Med 2004 27 5 471 476 15556746 10.1016/j.amepre.2004.08.010 PMC7134882

[B10] Armitage P Berry G Matthews JNS Statistical Methods in Medical Research 2002 4 Blackwell Science, Ltd., London, England

[B11] Hollander M Wolfe DA Nonparametric Statistical Methods 1999 2 John Wiley & Sons, Inc., New York, NY

[B12] Finer LB Zolna MR Unintended pregnancy in the United States: incidence and disparities, 2006 Contraception 2011 84 5 478 485 10.1016/j.contraception.2011.07.013 22018121 PMC3338192

[B13] CDC Sexually Transmitted Diseases Surveillance, 2008 2009 Centers for Disease Control and Prevention, Atlanta, GA

[B14] Hipwell AE Stepp SD Keenan K Chung T Loeber R Brief report: Parsing the heterogeneity of adolescent girls' sexual behavior: Relationships to individual and interpersonal factors J Adolesc 2010 34 3 589 592 20359740 10.1016/j.adolescence.2010.03.002 PMC2896989

[B15] Valois RF Oeltmann JE Waller J Hussey JR Relationship between number of sexual intercourse partners and selected health risk behaviors among public high school adolescents J Adolesc Health 1999 25 5 328 335 10.1016/S1054-139X(99)00051-8 10551663

[B16] Donnelly J Goldfarb ES Ferraro H Eadie C Duncan DF Assessing sexuality attitudes and behaviors and correlates of alcohol and drugs Psychol Rep 2001 88 3 Pt 1 849 853 11508032 10.2466/pr0.2001.88.3.849

[B17] Sabia JJ Rees DI The effect of sexual abstinence on females' educational attainment Demography 2009 46 4 695 715 10.1353/dem.0.0072 20084825 PMC2831358

[B18] Adolescent Health Care, Sexuality and Contraception http://www.aafp.org/online/en/home/policy/policies/a/adol3.html

[B19] AAP American Academy of Pediatrics: Committee on Psychosocial Aspects of Child and Family Health and Committee on Adolescence. Sexuality education for children and adolescents Pediatrics 2001 108 2 498 502 11483825

[B20] APHA Sexuality Education As Part Of A Comprehensive Health Education Program in K-12 Schools 2005 American Public Health Association, http://www.apha.org/advocacy/policy/policysearch/default.htm?id=1304

[B21] Emans SJ Brown RT Davis A Felice M Hein K Society for Adolescent Medicine Position Paper on Reproductive Health Care for Adolescents J Adolesc Health 1991 12 8 649 661 1839215 10.1016/1054-139x(91)90014-o

[B22] Santelli J Ott MA Lyon M Rogers J Summers D Abstinence-only education policies and programs: a position paper of the Society for Adolescent Medicine J Adolesc Health 2006 38 1 83 87 10.1016/j.jadohealth.2005.06.002 16387257

[B23] King SC Frank E The Unwillingness of Future U.S. Physicians to Limit Adolescent Prevention Counseling to Abstinence-Only Messages J Pediatr Adolesc Gynecol 2010 23 4 237 241 10.1016/j.jpag.2010.01.004 20382053

[B24] Croft CA Asmussen L A developmental approach to sexuality education: implications for medical practice J Adolesc Health 1993 14 2 109 114 10.1016/1054-139X(93)90094-6 8476873

[B25] Cuff PA, Vanselow NA Improving Medical Education: Enhancing the Behavioral and Social Science Content of Medical School Curricula 2004 Institute of Medicine of the National Academies, Washington, DC 20669422

[B26] Tobacco Use and Dependence Guideline Panel Treating Tobacco Use and Dependence: 2008 Update 2008 US Department of Health and Human Services, Rockville, MD

[B27] Bourgeois JA Ton H Onate J McCarthy T Stevenson FT Servis ME Wilkes MS The doctoring curriculum at the University of California, Davis School Of Medicine: leadership and participant roles for psychiatry faculty Acad Psychiatry 2008 32 3 249 254 10.1176/appi.ap.32.3.249 18467484

[B28] Keefe CW Thompson ME Noel MM Medical students, clinical preventive services, and shared decision-making Acad Med 2002 77 11 1160 1161 10.1097/00001888-200211000-00026 12431938

